# Microbial metabolites as engines of behavioral variation across animals

**DOI:** 10.1080/19490976.2025.2501191

**Published:** 2025-05-13

**Authors:** Eric Siaw Ntiri, Adam Chun Nin Wong

**Affiliations:** a Entomology and Nematology Department, University of Florida, Gainesville, FL, USA; b Genetics Institute, University of Florida, Gainesville, FL, USA

**Keywords:** Microbiome, metabolites, symbiosis, behavior, gut-brain axis

## Abstract

The microbiome, especially that present in the gut, has emerged as a key modulator of animal behavior. However, the extent of its influence across species and behavioral repertoires, as well as the underlying mechanisms, remains poorly understood. Increasing evidence suggests that microbial metabolites play an important role in driving behavioral variation. In this review, we synthesize findings from vertebrates to invertebrates, spanning both model and non-model organisms, to define key groups of microbial-derived metabolites involved in modulating seven distinct behaviors: nutrition, olfaction, circadian rhythms, reproduction, locomotion, aggression, and social interactions. We discuss how these microbial metabolites interact with host chemosensory systems, neurotransmitter signaling, and epigenetic modifications to shape behavior. Additionally, we highlight critical gaps in mechanistic understanding, including the need to map additional host receptors and signaling pathways, as well as the untapped potential of microbial biosynthetic gene clusters as sources for novel bioactive compounds. Advancing these areas will enhance understanding of the microbiome’s role in behavioral modulation and open new avenues for microbiome-based interventions for behavioral disorders.

## Introduction

From Pavlov’s classic experiment on conditioned reflexes to von Frisch’s characterization of the honey bee’s waggle dance, understanding the drivers of behavior has long been a central pursuit in biology. Historically, research has centered on the interplay between genes and external environmental factors. However, our understanding is evolving with the recognition that the pathways leading from genes to behavior can be modified by microbial communities residing within organisms, and new evidence suggesting that how animals perceive and respond to environmental stimuli could be molded by their microbial experience.

As our appreciation of the microbiome-behavior connection deepens, researchers are increasingly focusing on unraveling the chemical communications that underline microbiome-mediated behavioral modulation. Microbes possess biosynthetic gene clusters capable of producing a remarkable diversity of bioactive metabolites.^1–3^ Interestingly, many of these gene clusters are silent under standard laboratory growth conditions,^4–6^ signifying a vast untapped potential for discovering bioactive compounds.

Microbiomes associated with animal hosts can produce metabolites *de novo* or make use of host resources. These metabolites can be synthesized independently or through synergistic interactions between intra-kingdom species (e.g., bacteria-bacteria) or inter-kingdom species (e.g., bacterial-fungi) interactions.^4,5,7–9^ For instance, microbes can ferment host-derived carbohydrates and proteins to generate key metabolites such as short-chain fatty acids (SCFAs) and branched-chain fatty acids (BCFAs), which are integral to metabolic and immune functions.^10–12^ Additionally, some microbes actively contribute to the biosynthesis of neurotransmitters and neuromodulators, including dopamine, norepinephrine, serotonin, gamma-aminobutyric acid (GABA), acetylcholine, and histamine.^13–16^ The vast diversity of microbial metabolites suggests that the microbiome exerts its effects on host behavior through multiple signaling pathways.

Given the complexity of mammalian systems, studies using simpler models can provide valuable mechanistic insights into microbiome-behavior interactions.^17^ Model organisms such as the *Caenorhabditis elegans* and *Drosophila melanogaster* have been instrumental in uncovering fundamental biological principles that later translated into mammals, including the roles of Toll-like receptors in innate immunity,^18^ microRNA-mediated post-transcriptional gene regulation,^19,20^ and the molecular mechanisms of circadian rhythms.^21,22^ Their relatively simple nervous systems, combined with well-characterized microbiomes and genetic tractability, facilitate precise dissection of microbial metabolites effects on host behavior. Other non-mammalian systems, including certain insect and avian species, are also increasingly studied for their unique advantages. Some species offer closer physiological parallels to mammals,^23^ while others allow high-throughput behavioral assays in ecologically relevant contexts.^24^ Although findings from non-mammalian models must be extrapolated cautiously, given key biological differences such as the lack of adaptive immunity in invertebrates and variations in behavioral complexity, integrating findings across diverse systems offers complementary perspectives in understanding microbiome-mediated behavioral modulation.

**Figure 1.**
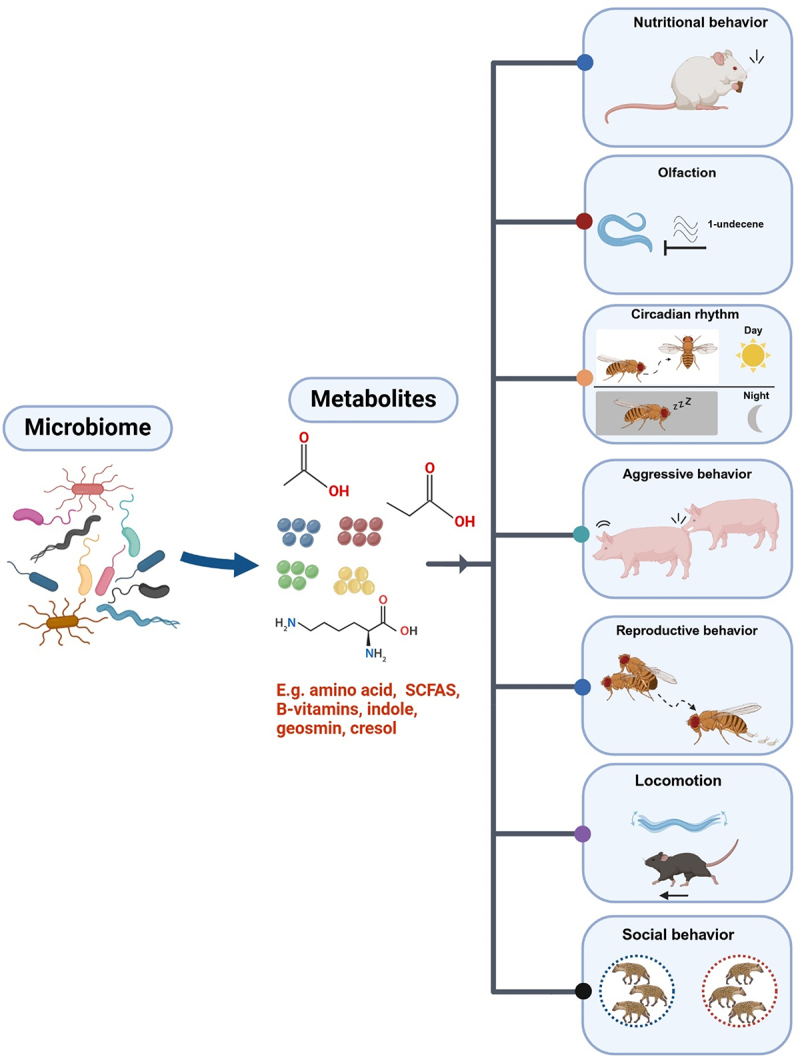
Microbial metabolites influence diverse host behaviors. This schematic highlights the interplay between microbiome-derived metabolites and host behaviors, illustrating the broad impact of microbial metabolism on behavioral modulation. Created in https://BioRender.com.

In this review, we synthesize evidence on how bacterial and fungal bioactive compounds modulate behaviors including feeding and foraging, olfaction, circadian rhythms, reproduction, locomotion, aggression, and social interactions (Figure 1). We also discuss mechanisms by which microbial metabolites interact with host chemosensory systems, neurotransmitter signaling, and epigenetic modifications to alter behavior, and identify key areas for future research.

## Nutritional behaviors

The need for nutrients to support reproduction, growth, and development manifests in various nutritional behaviors, including foraging and feeding.^25–29^ These behaviors have long been studied through the frameworks of optimal foraging theory (OFT) and nutritional ecology, which seek to explain how animals navigate complex dietary landscapes to optimize fitness.^30^ OFT conceptualizes foraging as an economic decision-making process, balancing the energetic costs and benefits of food acquisition, while nutritional ecology, particularly the geometric framework model, emphasizes the role of macronutrient balance in shaping dietary choices. These models are incomplete without considering the microbiome’s role.

One of the most direct ways the microbiome shapes nutritional behavior is through nutrient provisioning. Microbes can serve as direct food sources for certain animal species,^31–33^ as seen in the nematode *C. elegans* consuming bacteria from the phyla Proteobacteria, Bacteroidetes, Firmicutes, and Actinobacteria^34^ and in *Drosophila* that feed on various yeast species.^35^ Beyond being a passive food source, the microbiome actively contributes to the host’s metabolic profile by synthesizing or transforming nutrients. For example, the gut bacterium *Acetobacter orientalis* provides branched-chain amino acids such as leucine and isoleucine, which are important for larval growth in *Drosophila* .^36^ The gut microbiome also supplies B-vitamins, particularly riboflavin and thiamine, to support fly development under nutrient-limited conditions.^37,38^ Other invertebrate examples include gut bacteria *Enterobacteriaceae* in the Mediterranean fruit fly that can fix atmospheric nitrogen,^39^ *Blattabacterium* in cockroaches^40^ and the microbiome in herbivorous turtle ants^41^ that recycle nitrogen from urea and uric acid to meet the hosts’ nitrogen needs. In mice, *Lactobacillus intestinalis* converts dietary vitamin A into retinoid metabolites.^42^ Separately, in rats, high levels of vitamin A reduced sucrose preference and food intake.^43,44^ As retinoid signaling can influence appetite regulation,^45^ microbial metabolism of vitamin A may play a role in shaping feeding behavior. In humans, a comprehensive genomic analysis revealed that 40–65% of common gut bacteria possess biosynthetic pathways for essential B-vitamins.^46^ The analysis suggests that the gut bacteria engage in “cross-feeding”, whereby species that lack the ability to synthesize certain B vitamins rely on those produced by others. The human gut microbiome also consists of amino acid auxotrophies that depend on the amino acids synthesized by other members.^47^ These cooperative exchanges highlight the complexity of microbial community interactions in shaping the host’s overall nutrient balance.^48–50^

Despite the growing understanding of microbial contributions to host nutrition, evidence linking microbial nutrient provisioning to host feeding behavior remains scarce. Notable insights were yielded from studies on *D. melanogaster*^51^ demonstrating that dietary deprivation of any single essential amino acid (eAA) triggered a stronger appetite for amino acid-rich food. The response was mitigated by the presence of the commensal bacteria *Acetobacter pomorum* and *Lactobacillus* species, without measurable changes in the host’s amino acid levels. This suggests that the gut bacteria influence feeding behavior through mechanisms beyond mere nutrient supplementation. Further research by^8^ uncovered that *Acetobacter* uses lactate produced by *Lactobacillus* to generate essential amino acids. This cross-feeding enables *Lactobacillus* to persist in nutrient-imbalanced diets. Lactate was found to be necessary and sufficient for *Acetobacter* to alter the fly’s protein appetite, thus establishing a mechanistic link between microbial metabolic interactions and host feeding preference.

Besides nutrient provisioning, the microbiome influences nutritional behaviors by producing metabolites that interact with the host’s endocrine and nervous systems. For example, high-fat diet-induced obesity in female mice can be mitigated by a dietary intervention involving high-fiber inulin, which restructures the gut microbiome and increases short-chain fatty acid (SCFA) levels, leading to reduced food intake and ameliorated postpartum stress behaviors.^52^ In honey bees, conventional bees exhibited significantly higher sensitivity to sucrose compared to germ-free bees, a phenomenon potentially linked to differential SCFA levels and insulin signaling.^53^ Oleoylethanolamide, a metabolite partially synthesized from dietary oleic acid by gut microbes, regulates satiety and energy balance in mice by activating intestinal G protein-coupled receptor 119 and peroxisome proliferator-activated receptor alpha (PPAR-α).^54,55^

Some microbial metabolites shape nutritional behaviors by modulating gut hormones (Table 1). In mammals, enteroendocrine cells produce hormones like peptide YY (PYY) and glucagon-like peptide-1 (GLP-1) that communicate the body’s nutritional status to the nervous system, thereby regulating appetite and satiety.^67,68^ SCFAs such as butyrate, propionate, and acetate have been shown to impact the production of these gut hormones. For example, the administration of SCFAs in rodents increases PYY and GLP-1 levels,^56–58^ and similar effects have been observed in pigs administrated propionate.^59^ Microbially-derived tryptophan catabolites, such as indole, have been shown to simulate GLP-1 production, contributing to appetite suppression.^60,61^ Secondary bile acids derived from microbial processing of primary bile acids^69^ have also been shown to regulate postprandial GLP-1 secretion. In microbiome-depleted (germ-free or antibiotic-treated) mice, fasting levels of secondary bile acids including ω-muricholic acid (ωMCA), hyocholic acid (HCA), deoxycholic acid (DCA), and lithocholic acid (LCA) were reduced in the ileum, coupling with an impaired GLP-1 response to oil intake. Interestingly, supplementation with ωMCA and HCA directly stimulated GLP-1 secretion via the TGR5 receptor in microbiome-depleted mice and primary ileal cell cultures. By contrast, DCA failed to induce GLP-1 in ileal cells from microbiome-depleted mice, implying its effect on GLP-1 is microbiome-dependent.^62^

**Table 1.** Gut hormones in mammals and their insect homologs implicated in nutritional behavior and the microbial metabolites that modulate their release.

Mammalian gut hormones/peptides	Insect gut hormones/peptides	Microbial metabolites that modulate gut hormone release	Refs
Glucagon-like peptide-1	Adipokinetic hormone	SCFAs (acetic, propionic, butyric acids)	^56–59^
		Tryptophan catabolites (e.g. indole)	^60,61^
		Secondary bile Acids (deoxycholic acid, lithocholic acid)	^62^
Peptide YY	Neuropeptide F Short neuropeptide F	SCFAs (acetic, propionic, butyric acids)	^56–59^
Serotonin	Serotonin	SCFAs (acetic, propionic, butyric acids)	^63,64^
Cholecystokinin	Sulfakinins	SCFAs (acetic, propionic, butyric acids)	^65^
Leptin	Unpaired 1 & 2	SCFAs (acetic, propionic, butyric acids)	^66^
Insulin	Insulin	SCFAs (acetate, malate)	^53^

Beyond the gut, metabolites impacting the host’s neural circuits play a significant role in regulating nutritional behavior.^70^ For example, administration of butyrate, propionate, and acetate, was shown to suppress food intake via the vagal afferents in a dose-dependent manner in mice.^71^ Muropeptides, components of bacteria cell wall,^72^ target brain neurons expressing the Nod2 receptor to control feeding behavior in mice. Older female mice (7–8 months) that lacked Nod2 consumed more food than their wild type counterparts.^73^

Collectively, these findings demonstrate that the gut microbiome not only supplements host nutrition by providing essential nutrients but also actively shapes nutritional behaviors through metabolic interactions acting on endocrine signaling and neural pathways. As the microbiome is plastic and individualized, its differences could contribute to variation in nutritional behaviors, such as dietary preferences and feeding habits, across individuals and species.

## Olfaction

Olfaction, the sensory process through which organisms detect and interpret volatile chemical signals from the environment, is a crucial modality that guides behaviors essential for ecological fitness, such as finding food, mates, nesting sites, and detecting threats.^74–78^ Olfactory-guided behavior can be innate or learnt, reflecting a complex interaction between genetic predisposition and environmental experience.^74^ Increasing evidence suggests that microbes can modulate host olfaction at multiple levels.^79–81^ Intrinsically, the microbiome can influence the structure and function of host olfactory tissues.^82,83^ For instance, germ-free mice exhibited a thinner cilia layer, reduced cellular turnover, and heightened neuronal responses to odorants in the olfactory epithelium, compared to conventional mice.^83^ These changes are associated with reduced expression of xenobiotic metabolizing enzymes. It was proposed that without microbial metabolites to compete, fewer enzymes are required to clear odorants, resulting in longer-lasting odorant responses. However, since germ-free mice lack both gut and nasal microbiomes, it remains unclear whether these effects are driven by the gut microbiome acting remotely via circulating metabolites, or nasal microbiome acting locally on the tissues. Further research is needed to distinguish these contributions.

Microbial metabolism of host-derived compounds can further influence olfactory perception and behavior. For example, inhibition of trimethylamine N-oxide (TMAO) production by gut microbes using the inhibitor iodomethylcholine (IMC) impaired mice’s ability to detect and respond to certain odors, demonstrated by delayed detection of hidden food and reduced interest in scents like tap water and almonds. IMC-treated mice also had difficulty distinguishing between familiar and novel social partners, leading to decreased social interactions.^84^ In honey bees, the gut microbe *Lactobacillus* regulates tryptophan metabolism to indole that activates the aryl hydrocarbon receptor, influencing bee olfactory learning and memory. Conventional bees showed a higher learning rate in the association of nonanol to sucrose reward, compared to antibiotic-treated and axenic bees.^85^

New evidence suggests that microbial effects on olfaction can manifest over longer periods, such as priming the olfactory system, leading to sustained changes in preferences and behaviors. In *Drosophila*, early-life exposure to specific gut symbionts, particularly *Acetobacter*, has been shown to enhance behavioral attraction toward the microbes, suggesting that microbial experience during development may play a key role in shaping host olfactory preferences.^86^ The effect might be attributed to exposure to acetoin produced by egg-associated microbes.^87^ Additionally, flies fed on indole-3-acetic acid produced by *Pseudomonas juntendi* showed higher attraction to ethanol but lower attraction to 1-octanol, highlighting the potential role of microbial metabolites in shaping olfactory preferences linked to addiction-related behaviors.^88^ In *C. elegans*, the gut bacterium *Providencia* produces tyramine, which is converted by a host enzyme into octopamine. This neurotransmitter then acts on the OCTR-1 receptor on ASH nociceptive neurons, modulating the worm’s aversive olfactory response.^89^

Microbes in the external environment also shape olfactory behavior through the release of volatile metabolites. For example, in *D. melanogaster*, the microbial metabolite geosmin is detected by a specific olfactory receptor (Or56a), triggering a robust avoidance response. This behavior is believed to be an evolutionary adaptation to avoid harmful microorganisms.^90^ Similarly, in *C. elegans*, toxic bacterial metabolites such as tambjamine YP1 and violacein prompt the nematodes to avoid areas where pathogenic bacteria are present, thereby enhancing their survival.^91^ 1-undecene, a volatile compound derived from *Pseudomonas aeruginosa*, acts as a pathogen‐associated molecular pattern that mediates aversive response in *C. elegans*. Additionally, the metabolite induces immune effectors specific to *P. aeruginosa* via the AWB olfactory neurons, suggesting dual functions of microbial metabolites in host defense.^92^

In contrast to mediating avoidance, some microbial metabolites act as attractants. Yeast-produced volatiles including ethanol, 1-propanol, acetic acid, ethyl acetate, ethyl hexanoate and 1-butanol, have been shown to attract tephritids and drosophilid flies.^93,94^ These compounds signal the presence of fermenting fruits, which are optimal sites for feeding and oviposition. In the oriental fruit fly *Bactrocera dorsalis*, the gut bacterium *Citrobacter sp*. produces 3-hexenyl acetate. This volatile compound serves as an oviposition attractant, guiding female fruit flies to fruits harboring the bacterium.^95^ In the red turpentine beetle *Dendroctonus valens*, the frass microbiome produces verbenone, which is higher in females and serves as a pheromone to attract males and thus coordinated attacks on pine trees.^96,97^ For bumble bees (*Bombus impatiens*), nectar-inhabiting bacterium *Asaia astilbes* and fungus *Metschnikowia reukaufii* produce various volatile compounds, such as alcohols, aldehydes, esters, isoprenoids and ketones, among which the bee shows a strong olfactory preference for 2-ethyl-1-hexanol that is highly produced by the bacterium. Interestingly, bees consumed more *Metschnikowia*-conditioned nectar than *Asaia*-conditioned nectar, suggesting the microbial metabolites elicit distinct olfactory and gustatory preferences.^98^ SCFAs produced by microbes are also known ligands of olfactory receptors^70^ . For instance, propionic acid can elicit opposing behaviors in closely related species, attracting *D. melanogaster* larvae while repelling *D. suzukii* larvae.^99^

While these examples illustrate the roles of individual microbes, interactions between microbial species can give rise to new metabolites that modulate olfactory behavior in unique ways. For instance, *D. melanogaster* prefers co-cultures of *Saccharomyces* and *Acetobacter* over the same species grown separately. This olfactory preference is driven by acetate and related volatiles produced when *Acetobacter* metabolizes ethanol derived from *Saccharomyces*. Such emergent properties of microbial community interactions, driven by metabolic exchanges,^7^ provide fascinating insights into how multi-species microbiomes influence olfactory behavior.

In summary, both intrinsic and external cues from microbial metabolite are important drivers of olfactory behavior. Microbial metabolites affect not only the structure of olfactory tissues but also modulate how animals perceive and respond to odors. Additionally, microbial volatile signals can function as semiochemicals driving behavioral variation in response to a changing environment. Outstanding questions remain regarding the role of microbiomes in different body compartments, whether in the gut, nasal cavity, or other tissues, in modulating olfactory behaviors. The potential of multi-species microbial interactions in generating novel chemicals that modulate olfactory responses also warrants further investigation.

## Circadian rhythms

Circadian rhythms represent one of the most finely orchestrated biobehavioral systems, governing the temporal organization of various physiological and behavioral processes, from sleep to cognitive functions and metabolic activities. These rhythms are synchronized with the 24-h light-dark cycle and are regulated by a network of a central clock in the brain and peripheral clocks in other tissues.^100,101^ While these clocks were traditionally thought to be influenced by genetic and environmental factors, recent research reveals the gut microbiome’s role in modulating circadian processes.^101–106^ Chronotypes, which describes variations in individual circadian expressions such as “morning”, “evening” and “neither” chronotypes,^107^ are emergent properties of the complex interactions involving the microbiome, diet, and the circadian system.^106^

Despite not being directly exposed to light, the gut microbiome demonstrates diurnal oscillations in both its composition and function. A study in mice found that about 60% of gut microbial species show rhythmic fluctuations, with certain microbial pathways involved in energy metabolism, DNA repair, and cell growth peaking during the dark phase, while others involved in detoxification, motility, and environmental sensing are more active during the light phase.^108^ Interestingly, microbial oscillations are tightly linked to the hosts' circadian rhythms. For instance, mice with mutations in core clock genes exhibited significant microbial community shifts coupled with the loss of rhythms in specific microbial taxa.^109^ While the exact mechanisms linking microbial rhythms and host circadian processes remain to be fully established, emerging evidence suggests that microbial metabolites are key mediators in this interaction.

Microbial SCFAs have been shown to modulate the entrainment of peripheral clock genes within tissues such as the liver and kidney in mice.^110^ The timing of SCFA exposure is crucial for the effect, signifying that the microbiome communicates with host tissues to synchronize peripheral clocks. Using mice intestinal epithelial organoids,^111^ demonstrated that specific microbial SCFAs induced circadian phase shifts in a concentration-dependent manner. The effect appears to involve histone deacetylase (HDAC) inhibition, implicating microbial metabolites in the modulation of host epigenetics as a novel mechanism for circadian rhythm regulation. In humans, a study examining the relationship among circadian rhythms, eating behavior, and gut microbes showed that microbial SCFA (acetate, propionate, and butyrate) levels decreased as the day progressed. These changes were associated with time-dependent shifts in microbial compositions influenced by eating behaviors such as meal timing and frequency.^112^ Moreover, butyrate supplementation was shown to upregulate circadian-clock genes (CRY1, CRY2, PER1, and BMAL1) and improve sleep quality in ulcerative colitis patients, highlighting its potential as an adjunct therapy for mitigating disease symptoms.^113^

In addition to SCFAs, the microbiome also regulates circadian rhythms through the production of other metabolites, such as polyamines and bile acids. Polyamines can influence the interactions between clock proteins.^114^ Perturbation in polyamine levels, such as feeding on a diet low in polyamines or antibiotic treatment, lead to changes in the hepatic circadian transcriptome in mice.^105^ Certain bacteria and fungi can produce melatonin, a hormone recognized for regulating sleep-wake cycles. Emerging evidence suggests that the gut microbiome may influence circadian rhythm through melatonin synthesis and metabolism, positioning it as a potential therapeutic target for dysbiosis and circadian rhythm disorders.^115^

Diet has long been recognized to influence circadian rhythms. For example, high-fat diets can disrupt circadian clock timing and alter the expression of key circadian genes.^116,117^ A review by^118^ highlights examples in which different dietary conditions (high/low fats, sugars, fibers) modulate the gut microbiome composition, which in turn affect circadian rhythms, supporting the role of gut microbiome as a transducer of dietary signals to regulate both circadian rhythms and metabolism. In this context,^119^ showed that microbial metabolism of bile acids impacts circadian rhythms. Specifically, they generated a mutant strain of *Bacteroides thetaiotaomicron* lacking the bile salt hydrolase (BSH) enzyme and colonized germ-free mice with either the mutant or wild-type strain. Mice colonized with the BSH-deficient bacteria had higher bile acid levels, gained less weight on a high-fat diet, and exhibited lower fat levels in blood and liver compared to those with the wild-type strain. These mice also exhibited a metabolic shift, favoring burning fat over carbohydrate for energy. The altered metabolism was coupled with differential expression of circadian rhythm genes in the distal ileum. Furthermore, unconjugated bile acids produced through microbial deconjugation of liver-secreted bile acids, such as deoxycholic acid and chenodeoxycholic acid, have been shown to increase the amplitude of clock genes including PER and CRY genes.^120^ The effects have been observed across multiple mouse tissues, including the ileum, colon, and liver. These findings suggest bile acids may act as chronobiological regulators, linking dietary inputs to circadian control.

In summary, although gut microbes do not directly perceive light, their composition and metabolic activities exhibit diurnal rhythms that not only synchronize with, but also modulate host circadian rhythms. Various microbial metabolites, including SCFAs, polyamines, bile acids, and melatonin, can act as biochemical mediators influencing circadian genes’ expressions, the entrainment of peripheral clocks, and circadian-related behavioral outputs such as sleep. These findings emphasize the microbiome’s role as an integral component of circadian regulatory systems.

## Locomotion

Locomotion is a highly coordinated process that integrates neural, motor, and metabolic systems, serving as the foundation for all other behaviors discussed in this review. Decline in locomotor function is a hallmark of aging, while abnormalities in locomotor behavior are prominent symptoms in a range of neurological diseases, such as Parkinson’s and Alzheimer's diseases. The microbiome has been implicated in influencing locomotion across various animal models.^121–125^ Though outcomes can vary depending on factors such as age, sex, and genetic background, research has begun to unravel specific microbial effectors and metabolites involved in modulating locomotion.

For example, maternal exposure to bacterial endotoxin lipopolysaccharide (LPS) during late pregnancy significantly alters locomotive response in rat offspring.^126^ Specifically, adult offspring from LPS-treated dams exhibited increased amphetamine (AMPH)-induced locomotion compared to those from saline-treated dams. This observation raises the possibility that prenatal exposure to bacterial endotoxins sensitize the dopaminergic system, as AMPH acts on dopamine signaling by promoting the release of dopamine and blocking its reuptake through inhibiting the dopamine transporter. In addition, germ-free mice administered with live *Lactobacillus plantarum* showed increased locomotor behavior associated with increased dopamine and serotonin levels in the striatum.^127^ These findings point to microbial interactions with neurotransmitter pathways as a potential mechanism underlying locomotion. Microbial modulation of host locomotion can also be mediated through gut hormone signaling.^125^ shows that mice treated with a broad-spectrum antibiotic cocktail exhibited reduced locomotor activity, associated with elevated levels of the gut hormone GLP-1. The reduced locomotion could be reversed by blocking the GLP-1 receptor or disrupting vagal signaling. Importantly, when germ-free mice were re-colonized with *Lactobacillus reuteri* and *Bacteroides thetaiotaomicron*, GLP-1 levels were suppressed, and normal locomotor activity was restored, demonstrating that these specific gut microbes regulate locomotion through GLP-1 signaling and vagal pathways.

Several metabolic cues or mechanisms underlying microbial effects on locomotion have been identified. In female *D. melanogaster*, the gut bacterium *Lactobacillus brevis* was shown to influence locomotive behavior through modulating host sugar levels with the bacterial enzyme xylose isomerase.^122^ The study further pinpointed octopamine as a key player by showing that thermogenetic activation of octopaminergic neurons or administering octopamine suppressed the locomotion-modulating effects of xylose isomerase.

In *C. elegans*, feeding with the breast milk-derived *Lacticaseibacillus rhamnosus* has been shown to enhance locomotion and slow the decline in muscle function, while also significantly extending lifespan. The effect is associated with increased levels of specific bacterial metabolites, including sphingolipids, galactose, amino acids and fatty acids, and involves central stress-response (p38 MAPK) and insulin (daf-2) pathways.^128^

The locomotion-stimulating effects of microbial-derived propionic acid have also been shown in rats.^129^ demonstrated that intraventricular infusions of propionic acid significantly increased locomotion associated with neuroinflammation and oxidative stress in specific brain regions.^130^ showed that systemic administration of propionic acid at high concentrations led to conditioned taste and place avoidance in rats, indicating that this metabolite not only stimulates locomotion but also induces aversive behavioral responses. Conversely rats injected with a high dose of microbially-produced indole traveled shorter distance compared to control rats.^131^ Collectively, these examples illustrate how microbial metabolites can exert both stimulatory and inhibitory effects on locomotion, and how microbial metabolism can influence systemic metabolic states, which in turn affect behavior.

The relevance of microbial effects on locomotion and neurological disorders is suggested by a study by.^124^ In this study, mice transplanted with fecal microbiome from schizophrenia patients displayed schizophrenia-like behaviors, including hyper locomotion and impaired learning and memory. These behavioral changes were accompanied by disruptions in the kynurenine pathway linked to tryptophan metabolism. Specifically, the transplanted mice showed elevated levels of kynurenic acid, a metabolite that acts as an antagonist to N-methyl-D-aspartate (NMDA) receptors, potentially contributing to the observed behavioral abnormalities. This study raised the question of whether neurological disorders like schizophrenia may be transmissible through the microbiome and whether microbiome intervention can be applied to treat the disorder.

The therapeutic potentials of microbial compounds and metabolites in managing locomotion disorders have also been a subject of growing interest. Combined LPS and the anti-inflammatory drug indomethacin treatment was shown to significantly improve cellular repair and locomotor function in rats with spinal cord injury.^132^ Germ-free mice administered with microbial amino acids, SCFAs, and bile acids also showed an increase in skeletal muscle mass and improved muscle strength involved in locomotion, compared to untreated counterparts,^121,133^ pointing to microbial metabolites as potential therapeutic agents managing conditions like Duchenne muscular dystrophy.^133^

With accumulating evidence demonstrating that microbial metabolites such as SCFAs, indoles, sphingolipids, and amino acids can influence locomotion, current literature also highlights how factors like early-life exposure, antibiotic treatment, and fecal transplantation, can alter locomotor behavior by reshaping the microbiomes. These findings have important implications for understanding and potentially treating motor and neurological disorders. One striking aspect is that the same metabolites can elicit stimulating or inhibiting effects depending on the species or context. More work is needed to unravel the underlying mechanisms driving these divergent outcomes and behavioral variability.

## Reproductive behavior

Both the gut microbiome and microbiomes associated with reproductive organs (referred to as the “reproductive microbiome”) have been shown to affect reproductive physiology and behavior, including mating and sexual selection.^38,134–142^ While microbial influence on reproductive behavior is increasingly recognized, the underlying mechanisms are not yet fully understood.^136,143,144^ Recent studies have begun to elucidate the impact of microbial metabolites on reproductive health and behavior. For instance, the fungal metabolite zearalenone produced by *Fusarium* in animal feeds has been shown to promote reproductive organ growth in pigs, increasing the weight of reproductive tracts and sizes of the uterine and vulvar areas.^145^ In *Trichuris* nematodes, reduced levels of microbial-derived arginine were associated with decreased reproductive capacity.^146^ In giant pandas, metabolites such as acetic acid and 1-hexanol alcohols produced by gut bacteria *Veillonellaceae* and *Lactobacillilaceae* are speculated to serve as precursors for the synthesis of sex pheromones in the perianal glands, influencing mate selection and mating behavior.^147^

In *C. elegans*, the fungal metabolite 2,5-diketopiperazine gliotoxin, produced by *Penicillium, Myrothecium*, and *Aspergillus*, induce egg-laying behavior by targeting the serotonin receptors.^148^ The synergistic production of lactate and isoleucine by the *Drosophila* gut microbes *Acetobacter pomorum* and *Lactobacillus plantarum* increased female oviposition.^8^ Also, production of lactate by *Enterococcus* ameliorated the negative effect alkaline environment had on the ovipositional choices of *Drosophila*.^149^ The importance of microbiome on sex-specific reproductive behavior is further illustrated by studies on *D. suzukii*, where axenic female flies show reduced foraging activity and oviposition compared to conventionally reared flies.^150^ The behavioral effect is likely associated with the lack of bacterial-derived vitamin B2 that is crucial for oogenesis in flies.^150,151^

Taken together, studies in model organisms consistently show that axenic hosts show declined reproductive capacity, highlighting the microbiome’s role in reproductive fitness. Microbial metabolites can influence host reproductive behavior through multiple routes, by promoting the growth of reproductive organs, serving as sexual pheromones, and impacting the pathways governing mating and oviposition behaviors.

## Aggression

Studies on animal models including *Drosophila*, mice,^152–154^ and chicken,^155–157^ have provided valuable insights into the role of microbiome in modulating aggressive behaviors. They have revealed that the presence or absence of the microbiome can lead to notable differences in aggressiveness. For example, in *Drosophila*, axenic males displayed reduced aggressive behaviors, such as fewer lunging attacks and prolonged fighting latency, compared to their conventionally raised counterparts. Axenic female flies also showed a decrease in head-butting frequency and took longer to initiate such aggressive actions when competing for resources like yeast food. These behavioral differences were linked to alterations in octopamine levels.^153^ Infection with *Wolbachia* has been shown to reduce aggressive behavior in *Drosophila* males, which is also associated with reduced octopamine levels.^158^

In pigs, tail-biting behavior, a form of aggression where pigs bite the tail of other and cause injuries, is more prevalent in individuals with a higher relative abundance of Firmicutes in their gut microbiome. These “biter” pigs also had lower levels of fecal SCFAs such as acetate, propionate, butyrate and valerate, but higher plasma SCFAs levels.^159^ Similarly, in chickens, dominant roosters that engaged in aggressive behaviors such as pecking or scratching other birds, had higher levels of microbial SCFAs, including acetic acid, propionic acid, butyric acid, and valerate acid than in less dominant roosters.^155^ Total microbial SCFAs and lactates were also found to be higher in the cecum of hens exhibiting high feather-pecking behavior than those with low feather-pecking behavior, whereas the levels of biogenic amines such as putrescine and cadaverine were inversely associated with the feather-pecking behavior.^160^ In rats, subcutaneous administration of propionic acid has been shown to induce stronger aggressive behavior 14 days post-administration.^161^ While the above examples suggest a positive association between microbial SCFA and various aggressive behaviors, interestingly, in humans, the levels of microbially-produced SCFAs were shown to be reduced in schizophrenia patients displaying aggressive behaviors.^162^

Another mechanism involved in modulating aggression behavior is nitrogen metabolism. Gut bacteria in rats and humans, such as *Escherichia coli and Lactobacillus*, have shown the capacity to produce nitric oxide (NO) from nitrate or nitrite^163,164^ and NO is involved in the display of aggressive behavior, particularly in male mice, through interactions with serotonin.^165^

To conclude, aggression often arises as a result of direct competition with others or in response to threats. This makes aggression behavior sensitive to acute environmental cues. It is inherently social, context-dependent, and variable even within a species. Microbial metabolites like SCFAs, nitric oxide, and biogenic amines can alter the expression of aggression, sometimes in contradictory ways. In some species, for example, SCFAs can promote aggression, whereas they are linked to reduced aggression or social withdrawal in others. Such variability underscores the context-dependent nature of the microbiome in modulating aggression.

## Social behavior

The relationship between the microbiome and social behavior is reciprocal. Social behavior can influence microbial exposure and acquisition, whereas the microbiome has been implicated in social behavior regulation.^166–168^ An example of microbiome-social behavior connection is implicated in hyenas, where bacterial communities in scent glands, rich in fermentative bacteria, correlate with the volatile fatty acid in their scent secretions that are involved in social communication and hierarchical structure.^169^ However, a subsequent study indicate that social rank does not predict microbiome composition in hyena, though there are notable sex differences in the anal scent glands of juvenile hyenas and age-related differences in the prepuce and rectal microbiome between juveniles and adults.^170^ In wild Verreaux’s sifaka lemurs (*Propithecus verreauxi*), social group membership is reflected in the microbiomes of individual. Groups with denser grooming networks have more homogeneous gut microbial compositions, while more gregarious adult individuals and those that scent-mark frequently have greater microbial diversity.^171^

Further supporting the link between microbiome and social behavior, recent studies have shown that gut-derived metabolites can impact social behaviors in various animals. For example, administration of ergothioneine, a metabolite produced by the gut bacterium *Lactobacillus reuteri*, reduced social avoidance behavior in rats.^172^ Injection of the SCFAs, including acetate, butyrate and propionate into the brains of germ-free male mice, resulted in a decrease in the display of social novelty (described as time expended to recognize a new conspecific female mouse) compared to controls injected with artificial cerebrospinal fluid.^173^ In additional to its role in olfaction mentioned earlier, trimethylamine N-oxide (TMAO) administrated to mice resulted in lowered social dominance and reduced sexual preference, associated with significant changes in abundance over 200 metabolites.^174^

Research has also linked microbial metabolites to social behaviors in the context of developmental disorders. For instance, an assessment of fecal metabolites from infants found an association between variations in the levels of microbial metabolites, especially higher lactate and lower SCFAs levels, and fewer autism-related behaviors, depicted by a lower score on the social responsiveness scale (a rating scale used to measure behaviors related to autism spectrum disorder (ASD), where higher scores show more ASD-related behavior.^175^

The impact of social environments on the microbiome and behavior has also been explored. Juvenile social isolation in mice was found to increase the level of propionic acid in the gut. Mice reconstituted with fecal microbiome from socially-isolated mice or grouped-house mice supplemented with propionic acid all exhibited anxiety behavior and social deficits, mirroring the effects of juvenile social isolation .^176^ Transplantation of specific gut bacteria *Clostridiales, Lachnospiraceae* and *Ruminococcaceae* to germ-free mice also induced social avoidance behavior in the recipient mice and the effect was associated with high production of the microbial metabolite, cresol.^177^ A study on wild pandas showed that the microbial composition of the pandas’ feces and scent marking sites showed varied correlation with various volatile compounds, including heptadecane, L-α-terpineol, nonanal, iso-isovelleral, decanal and metabolites such as 11β-hydroxyandrosterone, idebenone, glycerol-3-phosphate that were also found in pandas’ scent marks.^178^ Although the precise roles of these individual compounds in modulating social interactions have not been tested, they appear to be signatures of the pandas’ scent marks that function as cues for kin recognition and sex identification.^179,180^ In insects, metabolites directly produced by the microbiome or which production is mediated by the microbiome, such as cuticular hydrocarbons (CHC), function as aggregation pheromones or kin recognition signals.^181^ For instance, antibiotic treatment in leaf-cutting ants altered their microbial composition and CHC profiles, leading to the ants receiving higher attacks from nestmates.^182^ Similarly, in the termite, *Reticulitermes speratus*, members covered in different bacterial odors from another colony faced heightened aggression from nestmates.^183^

In summary, studies have highlighted the dynamic feedback loop between the microbiome and social behavior, where the microbiome both modulates social interactions and is shaped by the social environments of the individuals. Microbial metabolites such as SCFAs, ergothioneine, cresol, and cuticular hydrocarbons have been identified as drivers of kin recognition and hierarchical interactions within populations. More research is needed to elucidate the mechanisms underlying how microbial metabolites regulate social behavior and the extent to which social environments influence the microbiome composition during development and across lifespan.

## Conclusion and future directions

The microbiome’s influence on animal behavior through specialized microbial metabolites is increasingly recognized. These metabolites are engines of behavioral variation, helping hosts adapt to changing environments, but dysregulation can lead to aberrant behaviors and diseases. Despite recent advances, significant gaps remain in our understanding of the underlying mechanisms. Here, we highlighted several topics for future research.

1. A comprehensive understanding of the host receptors and pathways that interact with microbial metabolites is foundational for advancing this field.^184^ While several receptors, such as G protein-coupled receptors (GPCRs),^65,185,186^ aryl hydrocarbon receptor,^187,188^ pregnane X receptor,^189,190^ IL-10 receptor^191^ and peroxisome proliferator-activated receptor γ,^192^ have been linked to microbial modulation of host behaviors, many are yet to be discovered. A deeper understanding of these receptors and their associated pathways is crucial to advancing our comprehension of the microbiome’s impact on behavior.
2. The role of epigenetic mechanisms in mediating microbiome-induced behavioral changes. Epigenetic mechanisms such as DNA methylation, chromatin modeling, histone modification, and noncoding RNA are emerging as modulators of neuroplasticity and behavior.^193–195^ For instance, *Drosophila* exposed to a high-sugar diet showed transgenerational reduced sweet sensitivity. The effect is attributed to upregulation of histone methylation, H3K27me3, transmitted maternally.^196^ Also, in *C. elegans*, maternal exposure to pathogenic *Pseudomonas aeruginosa* resulted in progeny that avoided the pathogen, a behavior modulated by RNA-mediated regulation of gene expression.^197^ Microbial metabolites such as SCFAs function as modulators of the host epigenetic process.^198–203^ Microbiome-mediated choline metabolism has also been shown to affect DNA methylation and anxiety behavior in mice.^204^ Given the central role of epigenetics in behavioral plasticity, elucidating how the microbiome influences epigenetic pathways in behavioral modulation represents a major advancement in the field.
3. Unlocking the potential of biosynthetic clusters. The vast diversity of the microbiome and its untapped biosynthetic pathways present both challenges and opportunities for future research. Many microbial biosynthetic clusters are inactive under standard laboratory conditions, which limits our ability to assess their full biological potentials.^5^ Efforts to optimize and engineer microbial communities to activate these latent pathways could unlock a broader array of microbial metabolites that can modulate host behaviors. This would not only expand our knowledge of microbial contribution to behavioral variation but also offer opportunities to harness these metabolites as therapeutic agents for behavioral disorders.

## Data Availability

Data sharing is not applicable to this article as no new data were created or analyzed in this study.
